# Osimertinib: Another medication related to osteonecrosis of the jaws? A case report and literature review

**DOI:** 10.3389/fphar.2022.947947

**Published:** 2022-08-10

**Authors:** Feng Wang, Shengnan Wei, Zexuan Zhang, Yuan Zhang, Jingya He, Bin Sun

**Affiliations:** ^1^ Department of Oral and Maxillofacial Surgery, School and Hospital of Stomatology, Jilin University, Changchun, China; ^2^ School of Public Health, Jilin University, Changchun, China; ^3^ Department of Critical Care Medicine, The First Hospital of Hebei Medical University, Shijiazhuang, China

**Keywords:** medication-related osteonecrosis of the jaw (MRONJ), osimertinib (AZD9291), side effect, EGFR, VEGF

## Abstract

**Introduction:** Medication-related osteonecrosis of the jaw (MRONJ) is an oral complication in cancer patients being treated with either antiresorptives, mainly denosumab and bisphosphonates, or antiangiogenic drugs. Osimertinib is a third-generation epidermal growth factor receptor (EGFR)-tyrosine kinase inhibitor (TKI) for the treatment of patients with EGFR T790M advanced non-small-cell lung cancer (NSCLC). TKI-induced osteonecrosis of the jaw has been reported in recent years, but these cases almost occur in combination with bisphosphonates, and the data on MRONJ associated to osimertinib is scarce.

**Case report:** We reported a case of MRONJ associated only with osimertinib. A 69-year-old female patient with NSCLC developed MRONJ after 4 years of treatment with osimertinib. Six months ago, she felt persistent pain and swelling in the right maxilla. After 3 months of pain, her dentist extracted one tooth in the right maxilla under local anesthesia. We examined her gingiva and found fistula and pus spillage. A digital volume tomography scan revealed sequestrum. The patient underwent surgical debridement of the necrotic bone under general anesthesia and administered intravenous antibiotics at the hospital. Histopathological analysis of the bone biopsy revealed a diagnosis of MRONJ.

**Conclusion:** This report provides evidence that osimertinib monotherapy can cause MRNOJ, and has a contribution to explore the formation mechanism of MRONJ. For those patients who take osimertinib, routine oral examinations and monitoring should be performed before and during treatment, as well as prompt closure of wounds and antibiotic treatment to avoid infection after invasive oral surgery such as tooth extraction.

## Introduction

Medication-Related Osteonecrosis of Jaws (MRONJ) is a complication of maxillofacial bone tissue necrosis that can be caused by taking systemic drugs, including bisphosphonates (BPs), used for osteoporosis or malignancy, and non-BPs, such as receptor activator of nuclear factor kappa-B ligand (denosumab), antiangiogenic drugs (bevacizumab), and IL-6 receptor antagonists (tocilizumab) (Khan et al., 2015; He et al., 2020; Sakkas et al., 2021). A diagnosis of MRONJ should be considered when patients present with all three of the following criteria: 1) current or previous treatment with antiresorptive or antiangiogenic agents; 2) exposed bone or bone that can be probed through an intraoral or extraoral fistula in the maxillofacial region that has persisted for longer than 8 weeks; and 3) no history of radiation therapy of the jaws or metastatic disease of the jaws (Ruggiero et al., 2014). Dentists play a pivotal role in preventing MRONJ; through thorough assessment, prophylactic dental treatment, and close multi-professional teamwork, the risk of MRONJ can be reduced. Therefore, the exploration of the etiology of MRONJ contributes to the early prevention and intervention.

Osimertinib is a third-generation epidermal growth factor receptor (EGFR) tyrosine kinase inhibitor (TKI), which is highly selective for EGFR*-*activating mutations and the EGFR T790M mutation in patients with advanced non-small-cell lung cancer (NSCLC) (Leonetti et al., 2019). Of the known side effects of osimertinib, diarrhea was the most frequent toxicity (44%), followed by rash (42%), dry skin (29%), and paronychia (25%) (Yi et al., 2019). Serious adverse events reported in 2% or more of patients were pneumonia and pulmonary embolism. The combination of TKI with BPs has been reported to increase the risk of MRONJ (Bozas et al., 2010; Hoefert and Eufinger, 2010; Nicolatou-Galitis et al., 2013). Subramanian G et al. reported a case of osteonecrosis associated with osimertinib in 2019 (Subramanian et al., 2019). However, this report did not describe whether the patient had taken other drugs than osimertinib. Their report didn’t include the diagnostic criteria of MRONJ stated by the AAOMS, radiological results and histopathological results. The evidence that MRONJ was caused by osimertinib is insufficient. This case suggests that osimertinib might cause MRONJ.

## Case report

A 69-year-old female patient presented to the Hospital of Stomatology, Jilin University in March 2020 with persistent swelling and pain in the right maxilla which occurred 6 months previously. Three months after the pain, the first molar of right maxilla was extracted the under local anesthesia by her dentist. The pain did not eliminate but aggravate in the following 15 days. The patient’s general medical history revealed that she had been diagnosed with NSCLC in May 2013, which had been treated with 125 mg Icotinib Hydrochloride Tablets daily for the first 3 years and 80 mg of Osimertinib Mesylate Tablets daily for the last 4 years. No history of smoking or alcohol abuse was reported.

**FIGURE 1:**
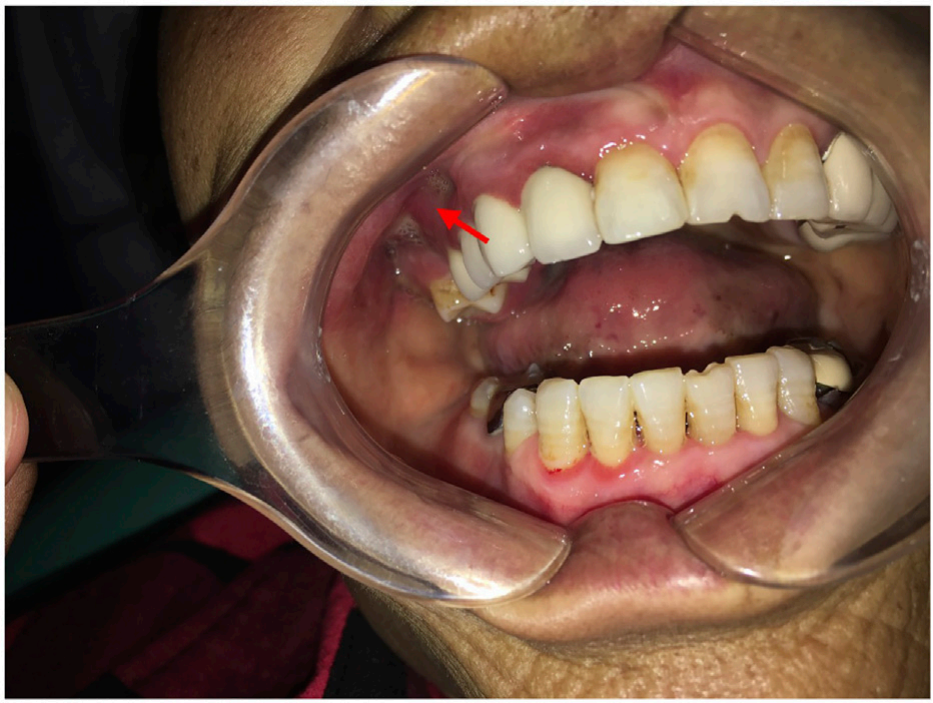
Intraoral picture shows swelling of the gingiva in the right maxillary molar area with fistula.

**FIGURE 2:**
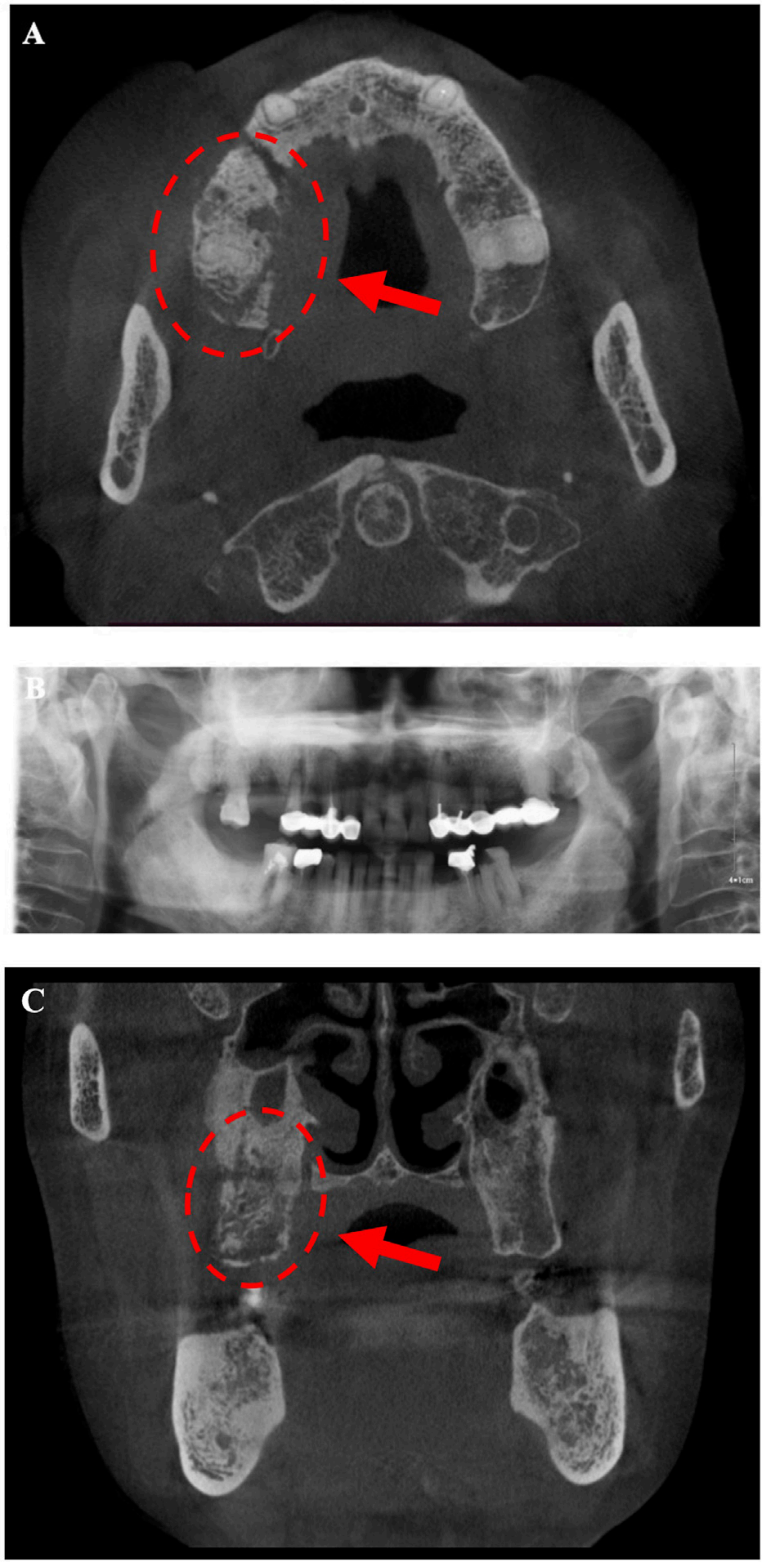
Radiograph **(A, B)** shows multiple teethmissing (teeth 14, 16, 26, 37,and 47) and osteonecrosis areas appear in the posterior part of the right maxilla. **(A, C)** The posterior part of the right maxilla presents a patchy bone destruction area with an unclear boundary. The cortical bone in the lesion area is discontinuous. The bone density of the lesion area is slightly higher than that of the contralateral bone, indicating sequestrum.

Intraoral examination revealed multiple missing permanent teeth (teeth 14, 16, 26, 37, and 47), swelling of the gingiva at teeth 14 and 16 with fistula, and overflow of pus when pressing (Figure 1). Extraoral, no lymphadenopathy or other alterations were evident. Panoramic radiograph and computed tomography (CT) showed teeth 14, 16, 26, 37, and 47 were missing, and the posterior part of the right maxilla presents a patchy bone destruction area with an unclear boundary. The cortical bone in the lesion area is discontinuous. The bone density of the lesion area is slightly higher than that of the contralateral bone (Figure 2). Based on the above symptoms, patient history, and medication history, we considered the bone lesion to be stage 2 of MRONJ based on the AAOMS severity staging system; the necrotic bone was exposed and infected as evidenced by pain and erythema in the region of the exposed bone with purulent drainage (Ruggiero et al., 2009).

**FIGURE 3:**
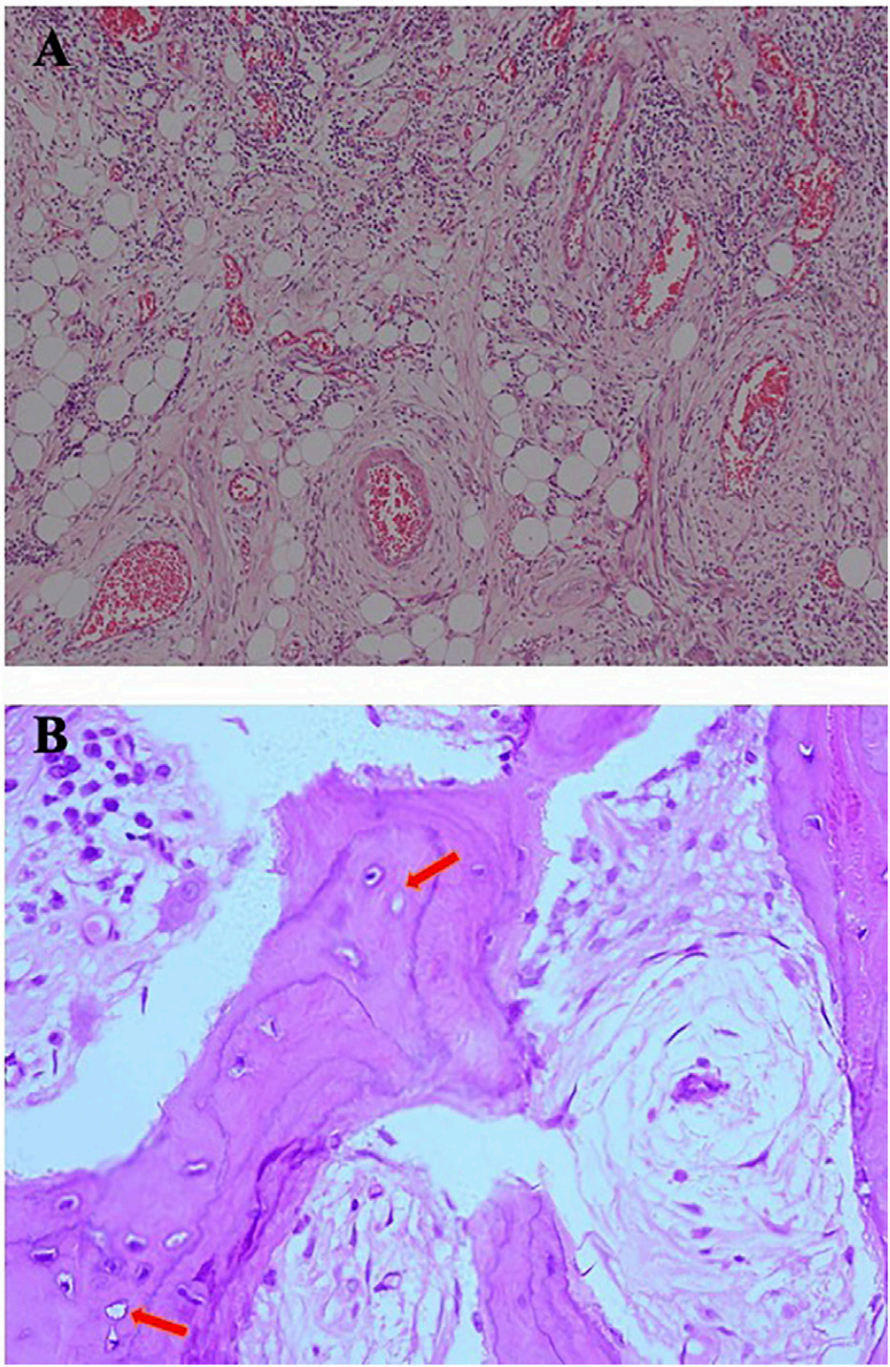
**(A)** Infiltration of plasma cells, lymphocytes, and neutrophils can be seen in fibrous connective tissue [hematoxylin and eosin (H&E), original magnification ×10]. **(B)** A large number of trabecular bone structures, sequestrum structures, reduced number of osteocytes, and empty lacunae (arrow) can be seen in some areas, which are signs of osteomyelitis (H&E, original magnification ×40).

**FIGURE 4:**
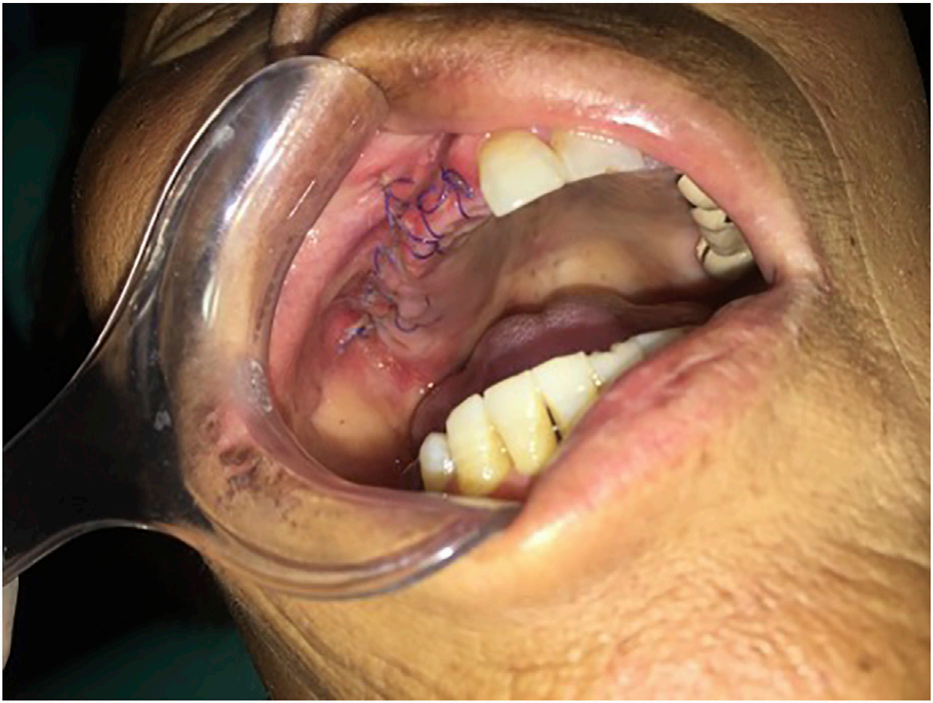
One week after surgery, the patient’s incision recovered well, with no swelling and oozing and no signs of infection.

Considering the role of infection in MRONJ (Otto et al., 2021), stroke-physiological saline solution and hydrogen peroxide irrigation (3%) were applied twice a day to clean patient’s wound to reduce infection. Five days later, the patient appeared in the operating room and underwent an osteotomy and extraction of the first molar under general anesthesia. Closed the wound with sutures after removal of the sequestrum and the patient received 2 days of intravenous cefazolin sodium pentahydrate 2.0 g (Shenzhen China Resources Gosun Pharmaceuticals Co., Ltd., China) twice a day. During hospitalization, the surgical site was treated with a povidone-iodine gargle (1%) under continuous antibiotic therapy. The specimens were sent for histological examination. Three days later, the pathological diagnosis was reported as osteomyelitis of the maxilla. As shown in Figure 3, there are some empty lacunae in the trabecular bone and a large number of lymphocytes and plasma cells in some areas, which are signs of osteonecrosis. One week later, the patient’s wound was healing well with no signs of infection (Figure 4).

## Discussion

At present, there are many hypotheses about the pathogenesis of MRONJ, among which angiogenesis inhibition is one of the most important mechanisms (AlDhalaan et al., 2020). Angiogenesis plays a crucial role in post-traumatic regeneration when defects occur in soft and hard tissues (Adams and Alitalo, 2007; Siemerink et al., 2010; Melincovici et al., 2018). Angiogenesis is a complicated process regulated by a variety of stimuli, some of which serve as proangiogenic agents and others as angiogenesis inhibitors (Rosen, 2002). VEGF is a proangiogenic factor that can promote mitosis and anti-apoptosis of endothelial cells, increase vascular permeability and promote cell migration. Because of these effects, it positively contributes to the regulation of normal and pathological angiogenesis processes (Melincovici et al., 2018). VEGF expression is driven by many factors, such as growth factors, cytokines, hypoxia, and solid malignant tumors (Olson et al., 1994; Joseph et al., 1997).

**FIGURE 5:**
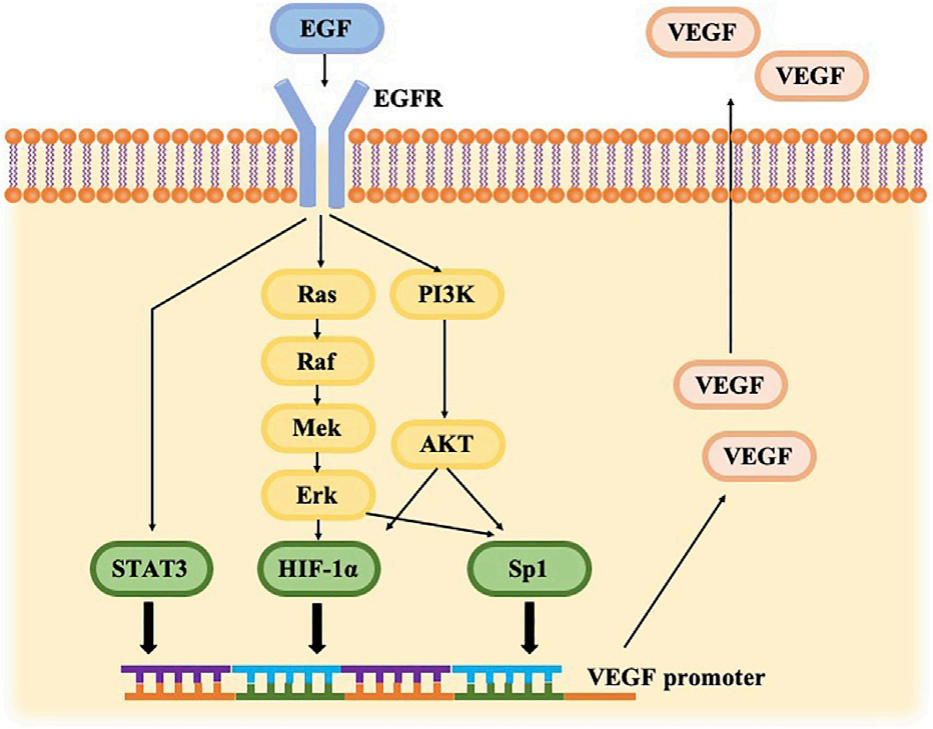
Cross-talk between the VEGF and EGFR pathways. EGFR signaling can trigger PI3K/AKT and RAS/RAF/ERK pathways, driving HIF-1α and Sp1 up-regulation. EGFR activation generates STAT3 directly. HIF-1α, Sp1, and STAT3 bind to THE VEGF promoter, resulting in VEGF gene expression.

EGFR, the first member of ErbB, is a single chain transmembrane polypeptide protein that is composed of an extracellular ligand-binding domain, a hydrophobic transmembrane region, an intracellular receptor tyrosine kinase (RAK) domain (Bessman et al., 2014). When epidermal growth factor (EGF) binds to the extracellular domain of EGFR, causing receptor dimerization, catalyzing tyrosine kinase activity, and inducing downstream signal cascades, such as mitogen-activated protein kinase (MAPK), phosphatidylinositol 3-kinase (PI3K)-Akt/PKB and the signal transducer and activator of transcription (STAT) pathways (Haley et al., 1989; Carpenter and Cohen, 1990; Larsen et al., 2011). Phosphorylation activation of Sp1 is mediated by MAPK and PI3K signaling pathways. STAT3 is a direct substrate of EGFR (Niu et al., 2002), and both Sp1 and STAT3 can directly bind to the promoter of VEGF to regulate the expression of VEGF (Larsen et al., 2011). Phosphorylation of Akt and Erk subsequently increased the expression of hypoxia inducible factors-1α (HIF-1α), which expresses VEGF on the transcriptional level *via* the hypoxia response element (HRE) (Pore et al., 2006). EGFR regulates VEGF expression *via* the MAPK and PI3K signaling cascades and at least three different transcription factors, STAT3, Sp1, and HIF-1α in a hypoxia-independent manner as illustrated in Figure 5.

Osimertinib, as an irreversible tyrosine kinase inhibitor, is approved as a therapy for advanced NSCLC with EGFR mutation. The acrylamide group of osimertinib covalently binds to C797 at the edge of the Adenosine triphosphate (ATP) binding site of the EGFR gene catalytic domain, thereby irreversibly binding to EGFR and competitively inhibiting downstream pathways (Cross et al., 2014; Zhang et al., 2016).

The VEGF pathway is one potential supplementary target of EGFR inhibition in NSCLC and the VEGF signaling pathway plays a crucial role in neoangiogenesis. HIF-1α, Sp1 and STAT3, which can bind to VEGF promoters, are constitutionally upregulated by EGFR activation in a hypoxia-independent manner (Xu et al., 2010), resulting in enhanced VEGF expression (Jackson et al., 2010). Different routes, including PI3K/AKT, MAPK, and STAT pathways, are blocked when the EGFR pathway is disrupted (Zhang et al., 2016), resulting in VEGF protein production inhibition and decreased angiogenesis (Ma et al., 2019), culminating in ischemia and eventually MRONJ.

We recognized several limitations of this case report. The patient had taken icotinib for 3 years before taking osimertinib. It was not known whether icotinib had an effect on the development of MRONJ. It is essential that further prospective studies with sufficient number of patients and standardized assessment protocols to confirm that osimertinib is associated with MRONJ.

This case provided important information that contributed to advances in the causes and prevention of MRONJ. Therefore, an important potential mechanism of osimertinib-related MRONJ is to reduce the expression of VEGF by inhibiting EGFR, thus causing angiogenesis disorders. After invasive oral treatment such as tooth extraction, local bone tissue is in a state of ischemia and wound closure is difficult due to angiogenesis disorder, resulting in prolonged wound recovery time and osteonecrosis of jawbone.

## Conclusion

In conclusion, although the mechanism of MRONJ is not completely clear, this case supplemented new underlying factors. Significantly, as infection is an important factor in MRONJ, systematic oral examinations and regular observation should be performed after osimertinib treatment to avoid apical periodontitis and periodontal infection. Invasive surgery such as tooth extraction can be performed, ensuring that proper wound closure techniques are performed as well as the application of appropriate antibiotics, thus reducing the risk of MRONJ occurrence. But when it comes to the treatment of MRONJ, it should be based on complete removal of the necrotic bone and wound closure. Dentists and oncologists should integrate and share information about the treatment and play their specific roles at each stage to prevent the occurrence or development of MRONJ and to accomplish individualized treatment. It has been shown that primary closure of the wound after extraction and prophylactic use of antibiotics could dramatically reduce the incidence of MRONJ (Saia et al., 2010; Hasegawa et al., 2017). For patients on long-term osimertinib, invasive procedures such as tooth extraction are possible, but require primary wound closure, and prophylactic use of antibiotics.

## Data Availability

The raw data supporting the conclusion of this article will be made available by the authors, without undue reservation.
